# Supplementary material for: Harmine Selectively Drives Human Beta Cell Differentiation and Function Via Protein Kinase A Pathways
Source: bioRxiv. 2025 Dec 8:2025.12.07.692818. Preprint. [Version 1] doi: 10.64898/2025.12.07.692818 (PMC12822811; doi:10.64898/2025.12.07.692818)
Supplement: Supplementary file 1 [file NIHPP2025.12.07.692818v1-supplement-1.pdf]

bioRxiv preprint doi: https://doi.org/10.64898/2025.12.07.692818; this version posted December 8, 2025. The copyright holder for this preprint (which was not certified by peer review) is the author/funder, who has granted bioRxiv a license to display the preprint in perpetuity. It is made available under a CC-BY 4.0 International license.

Supplementary Figures

Supplemental Fig. 1. Time Course of Increase in Selected Differentiation Markers. Note that PDX1, MAFA, NKX6.1 and C2CD4A rise within 2-8 hours, whereas MAFB, ENTPD3 and SLC2A2 (GLUT2) require 12-24 hours to respond. Note also that other transcription factors (NEUROD1, FOXO1, C2CD4B) show little response. Note that Insulin mRNA changes only modestly, as reported previously, although insulin secretion increased in vitro and in vivo in response to harmine[17,18]. Asterisks represent P<0.05 for the complete dataset vs. time 0 by one-way ANOVA, and the # symbols indicate the earliest time point at which the factor in question becomes elevated, as determined by paired T-tests vs. time 0.

Supplemental Fig 2. Time Course of Harmine and Forskolin Induction of ^{Ser133}P-CREB In Mouse R7T1 Cells. The point is that harmine, like forskolin, induces ^{Ser133}P-CREB phosphorylation in R7T1 cells, beginning within 30 min, and gradually declining over 2-3 hours, paralleling the effects of forskolin.

bioRxiv preprint doi: https://doi.org/10.64898/2025.12.07.692818; this version posted December 8, 2025. The copyright holder for this preprint (which was not certified by peer review) is the author/funder, who has granted bioRxiv a license to display the preprint in perpetuity. It is made available under a CC-BY 4.0 International license.

Supplementary Tables

Supplementary Table 1. Human Islet Organ Donor Summary.

Supplementary Table 2. Primers Used For PCR Experiments.

Supplementary Table 3. Human Islet Proteomic Data.

The Time Courses Of Beta Cell Transcription Factors And Phenotypic Genes Response to Harmine Is Rapid, and SLC2A2 Is Most Robust.

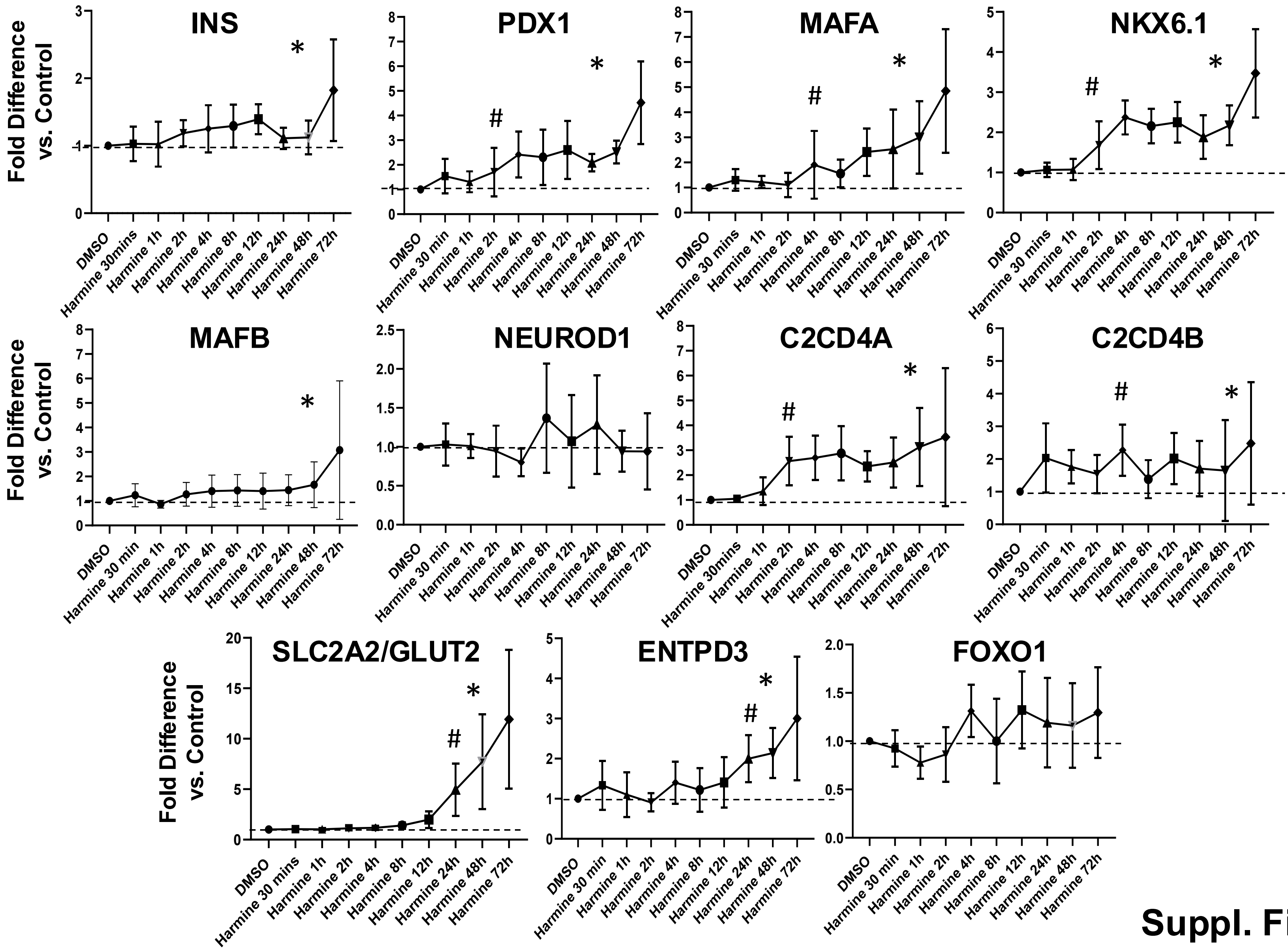

Suppl. Fig 1

The Time Course of Nuclear p-Ser133-CREB Appearance in R7T1 Cells Is Rapid

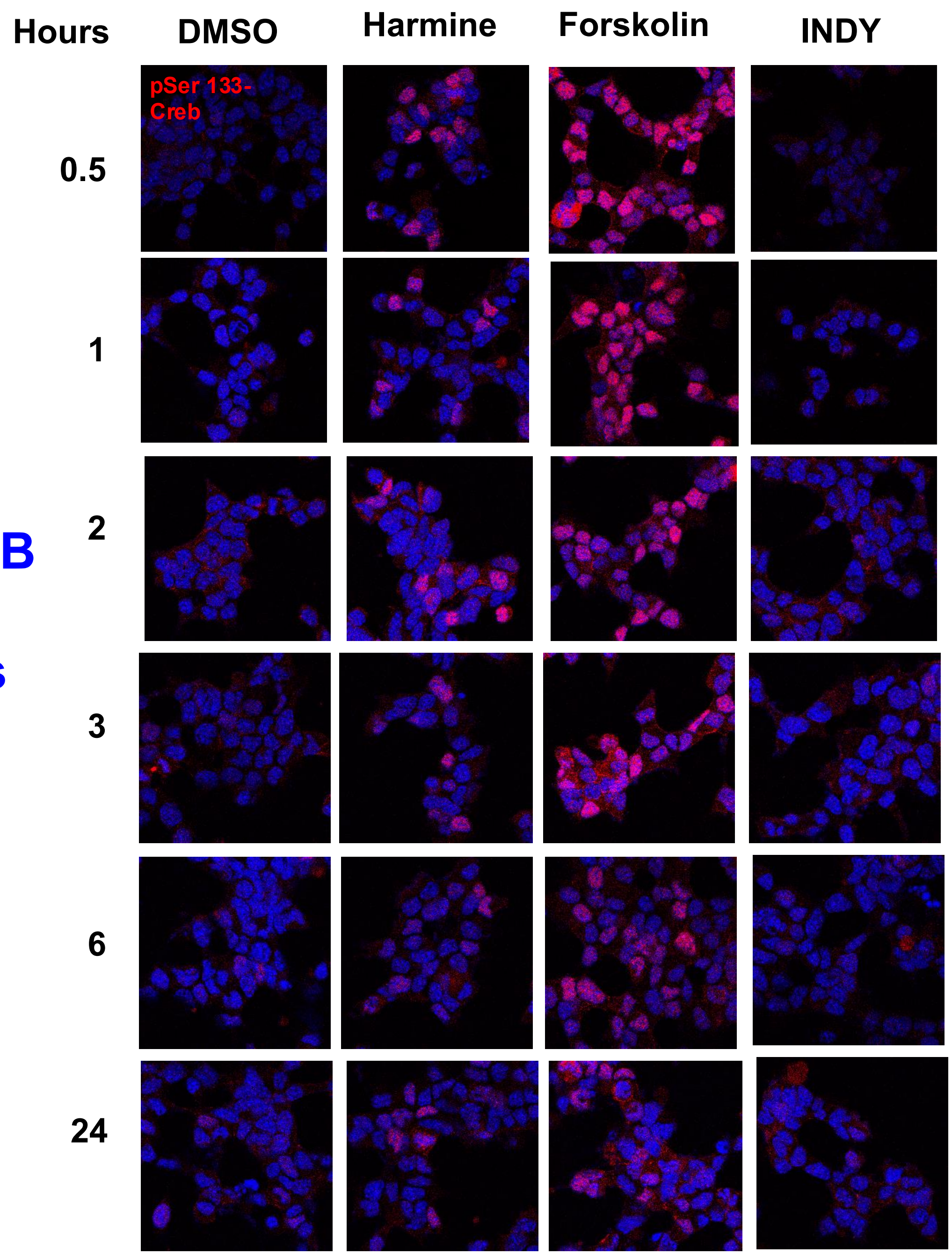

Suppl. Fig. 2
